# PCdare software registers 3D back surface with biplanar radiographs: application to patients with scoliosis

**DOI:** 10.3389/fdgth.2025.1682398

**Published:** 2025-12-10

**Authors:** Mirko Kaiser, Martin Bertsch, Christoph J. Laux, Sabrina Catanzaro, Tobia Brusa, Marco Wyss, Volker M. Koch, William R. Taylor, Saša Ćuković

**Affiliations:** 1 Laboratory for Movement Biomechanics, ETH Zurich, Switzerland; 2 University Spine Center Zurich, Balgrist University Hospital, University of Zurich, Zurich, Switzerland; 3 Biomedical Engineering Laboratory, Bern University of Applied Sciences, Biel, Switzerland

**Keywords:** 3D registration, biplanar radiographs, 3D surface scanning, automation, validation, scoliosis

## Abstract

Optical 3D surface scanning is used increasingly to assess spinal deformity of patients with scoliosis. However, approaches based on optical 3D scanning often underestimate the spinal deformity. To improve the accuracy of such estimates, deeper understanding is required of scoliosis and its effect on the back shape. We present the PCdare research software which registers 3D surface scans with the corresponding biplanar radiographs semi-automatically and facilitates investigations into the relationship between surface and internal modalities. PCdare revealed very strong correlations between the spinous process line and internal spinal alignment, and a median Cobb angle difference of less than 1° from the clinical gold standard. These results increase confidence in the use of 3D scanning with a “back-shape-to-spine” approach and confirm the applicability of PCdare to investigate the relationship between internal alignment and back shape in research.

## 1 Introduction

Adolescent idiopathic scoliosis (AIS) is by far the most common deformity of the spine and typically occurs in teenagers. If left untreated, AIS can induce cardiopulmonary impairment, cosmetic alterations, and discomfort (1). The current clinical gold standard for assessing AIS is radiography. Depending on the severity or risk of progression, patients with AIS undergo radiography every 6 months, resulting in a significant radiation burden in young individuals (2). Several studies have reported that the incidence of radiation-induced cancer types in patients with AIS is five times higher than in individuals without AIS (3, 4). Modern low-dose EOS systems do reduce radiation, but lifetime cancer risk remains non-zero, and further studies are recommended (5). As a result, optical 3D surface scanning is increasingly investigated and used in medical applications as a non-invasive and non-ionizing approach to screening in mostly young individuals (6–13).

Scoliosis is defined as a lateral curvature of the spine with a Cobb angle of 10 degrees or greater; the Cobb angle is the frontal angle between the two most tilted vertebrae. However, correlations between Cobb angles estimated solely from the 3D back surface in the “back-shape-to-spine” approach and Cobb angles obtained from radiographs remain moderate, with a tendency to underestimate the Cobb angle from optical 3D scanning (6). To improve the back-shape-to-spine approach and the algorithms that estimate internal spinal alignment (ISL), deeper understanding is required of scoliosis and its influence on the shape of the back. Bassani et al. (6) compared the Cobb angles obtained using DIERS formetric 4D (DIERS International GmbH, Schlangenbad, Germany) with the Cobb angles obtained using radiography (EOS imaging, Paris, France). They found only a moderate correlation between Cobb angles but did not investigate the underlying structure and its influence on the back shape. Cukovic et al. (14) investigated the relationship between internal spinal alignment and 3D back shape with data from 30 patients with AIS. The optical 3D surface scans were acquired as 3D point clouds with ARTEC Eva 3D (Artec 3D, Luxembourg) and the biplanar radiographic images with EOS imaging. The 3D back surface point clouds were manually registered with the radiographic images in CATIA v5-6R2013 (Dassault Systèmes, Vélizy-Villacoublay, France), a process that can take up to 5 min for a single registration. Beyond a certain volume of patient data in such research projects, manual registration is no longer feasible, and fully automatic registration is required.

To the best of our knowledge, no publicly available software currently provides fully automated registration of optical 3D surface scans with biplanar radiographic images and includes evaluations of internal alignment and back surface for research purposes. A system commonly used in clinics is a picture archiving and communication system (PACS). PACS enables clinicians to store and process radiographic images manually. Another free tool available used in research for annotating clinical parameters of the spine from radiographic imaging is Surgimap (Surgimap, Nemaris, New York, USA) (15). Surgimap enables the localization of each vertebra, including the evaluation of common clinical parameters such as Cobb angles. It includes a coronal and sagittal wizard that requires only a few minutes to annotate the entire spine in anteroposterior and lateral images; however, patients and images are managed manually, requiring substantial time. In addition, there is no option to upload 3D surface scans and therefore registration is not possible. Other semi-automatic and fully automatic computer-aided systems, including the sterEOS workstation (EOS imaging, Paris, France), methods, and algorithms have been proposed to estimate Cobb angles from radiographs (16–19) but these are limited to radiographs, provide no option to load 3D surface scans, and are not accompanied by publicly available code. Algorithms for the registration of 3D surface scans with 2D radiographs are available (20); however, most of the proposed 3D registration solutions focus on the registration of other modalities, such as CT with x-ray (21–23), CT with 3D scanning (24–26), and MRI with 3D scanning (27). Furthermore, the lack of publicly accessible code has led to a recurring scenario in which research groups must develop similar research software solutions independently, resulting in redundant effort.

In this paper, we present the research software PCdare, including publicly available code (see Data Availability Statement). PCdare enables semi-automatic registration of 3D surface scan point clouds (PCs) with the corresponding biplanar radiographs and thus represents a first step towards full automation. The PCdare software can be used in research to evaluate the relationship between the shape of the back and the internal alignment of the spine in patients with scoliosis. Furthermore, PCdare has been validated by comparison with the current gold standard in clinical practice.

With PCdare, the correlation between external back shape and internal spinal alignment can be systematically investigated, forming the basis for future approaches that predict ISL from back shape using optical 3D scans. This could enable clinicians to reduce the frequency of radiographic imaging and rely more on non-ionizing methods for routine monitoring.

## 2 Materials and methods

**Figure 1:**
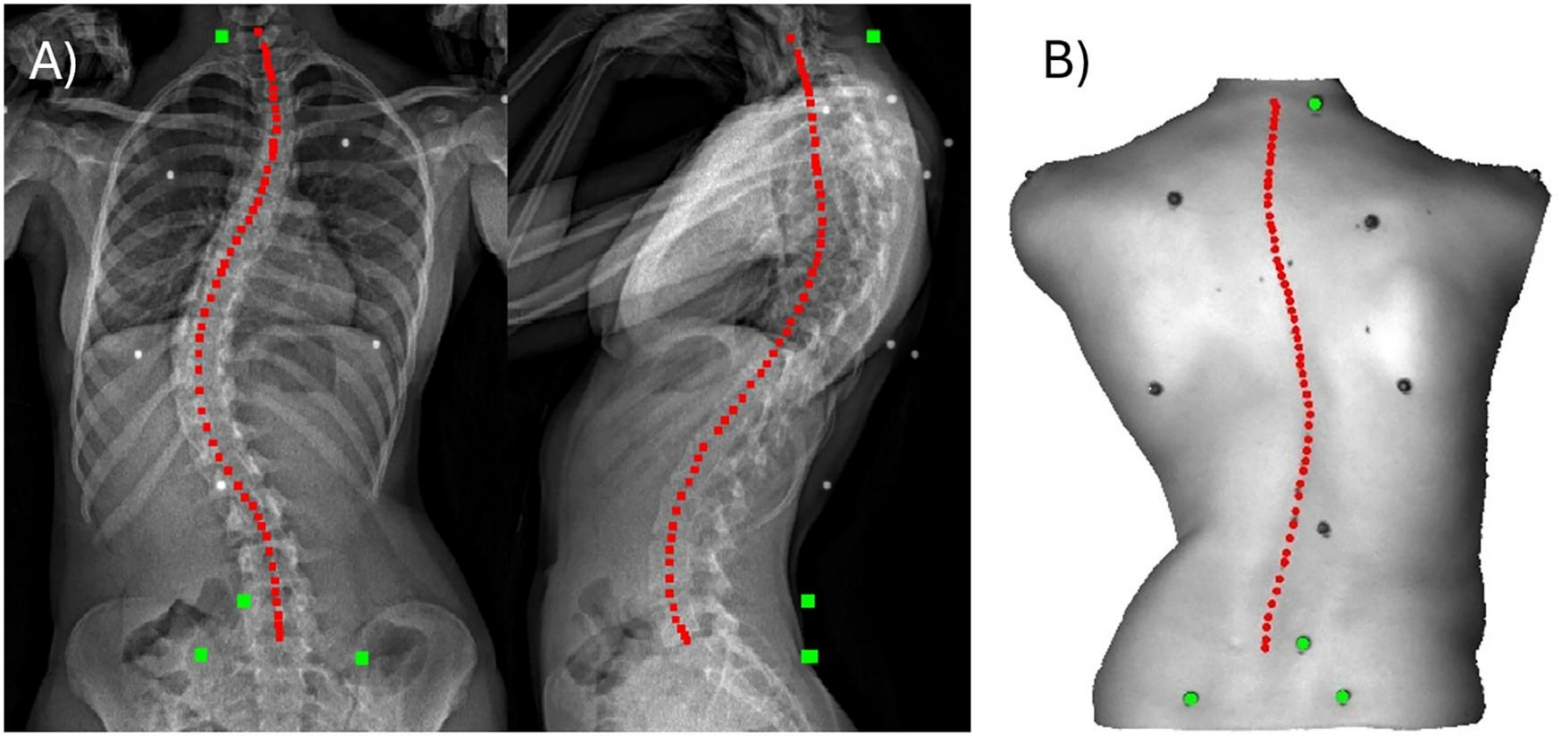
**(A)** biplanar 2D radiographic images with radiopaque spherical markers (white dots), marker selections (green) and line markings for the ISL (red) as displayed by the PCdare software in drawing mode. **(B)** Corresponding 3D surface scan as point cloud (3D PC) with radiopaque spherical markers (black dots), marker selections (green) and line markings for the SPL (red).

The PCdare software has been written in MATLAB R2021b (MathWorks, Natick, Massachusetts, United States) and can be used to semi-automatically register 3D surface scan point clouds with the corresponding biplanar radiographs: two 2D radiographic images (Figure 1A). This registration is necessary to evaluate the relationship between back shape and internal spinal alignment, in this work by examining the correlation between the spinous process line (SPL; Figure 1B) and the line through the centroids of the vertebral bodies, termed the internal spinal alignment (ISL; Figure 1A). Registration and evaluation involve the following steps. First, markers are selected, and line markings are drawn in both the 3D surface scan and the biplanar radiographs (subsection 2.1). Then the 3D surface scan and the biplanar radiographs are registered fully automatically (subsection 2.2), and SPL and ISL lines are generated from the line markings and smoothed. Finally, the correlations between SPL and ISL are calculated (subsection 2.3). Subsection 2.4 illustrates the practical value of the PCdare software in research by comparison with the current gold standard in clinical practice.

The PCdare software was used on data from 30 patients with AIS, collected at a single academic spine center [median Cobb angle of primary curve 29° (IQR 25°, range 9–54°); 18 years (IQR 5 years, range 11–25 years); BMI 19.4 kg/m^2^ (IQR 4 kg/m^2^, range 16–28 kg/m^2^); 18 females, 12 males]. Patients with scoliosis other than idiopathic, and those who had undergone bracing or surgery, were excluded (28, 29). The patients were equipped with nine radiopaque spherical markers with a diameter of 5 mm on anatomical landmarks (Figure 1B), and all spinous processes from C7 to L5 were marked with a pen. The patients then underwent biplanar radiography (EOS imaging) in an upright standing posture as part of their standard medical treatment. After the EOS imaging, the patients were taken to a consulting room; there, they replicated the upright posture using a pose frame (28, 29), and the back surface was scanned with a 3D surface scanner, a Photoneo MotionCam-3D (Photoneo s.r.o, Bratislava, Slovakia).

### 2.1 Methods of marker selection and line markings

Markers are necessary to register the 3D surface scan with biplanar radiographs (subsection 2.2), and line markings are necessary to calculate the correlation between SPL and ISL (subsection 2.3). The markers are selected, and line markings drawn with a touch pen on a laptop with touchscreen. When the PCdare software is in drawing mode, the user is automatically guided through all patient data to facilitate batch processing. The biplanar 2D radiographic images are displayed side by side for each patient (Figure 1A). For registration at least three markers in each image as far apart as possible in horizontal and vertical directions are required. In our study, we therefore used the four markers at C7, L5, and left and right posterior superior iliac spine (PSIS), which are often chosen in the literature (30, 31). Because marker selection was performed manually, only four markers were chosen instead of all nine available to reduce processing time. Next, the user draws the ISL from the centroid of C7 to the middle of the lower endplate of L5 in each image. Afterwards, the corresponding 3D point cloud (PC) is shown (Figure 1B). Again, the user selects the same markers on the 3D PC surface. Finally, the user draws the SPL from the C7 to the L5 markings. The 2D radiographic and 3D PC marker positions and raw line markings are immediately saved in a file in an output folder together with metadata, including timestamps, to allow verification of time requirements per patient.

### 2.2 Method of 3D registration

**Figure 2:**
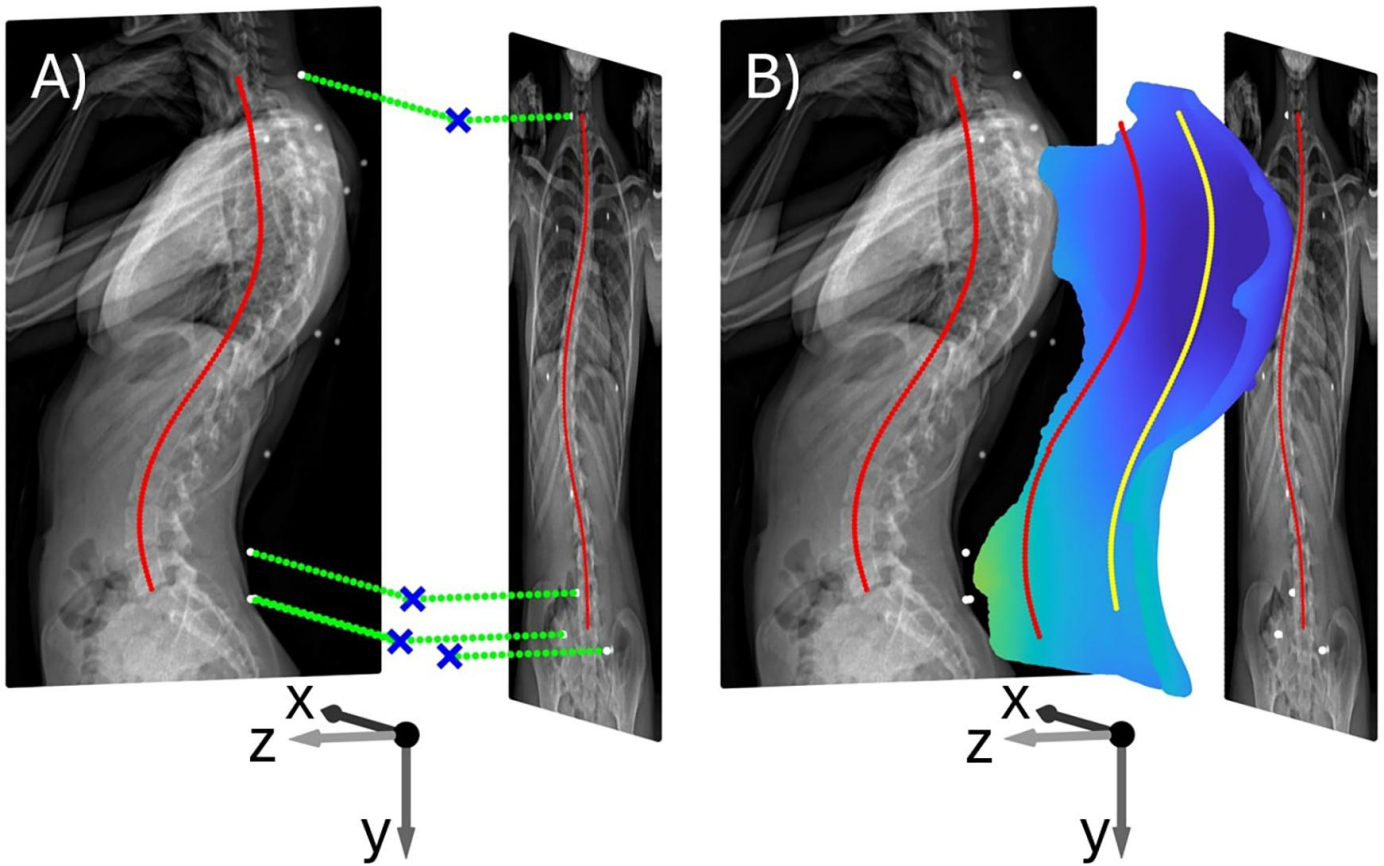
**(A)** biplanar radiographs with 3D marker positions (blue) from projections (green) of the 2D radiographic marker selections (white). **(B)** Biplanar radiographs with 3D back shape point cloud (blue), SPL (yellow) and ISL (red) after transformation into the EOS coordinate system (x,y,z).

To register the 3D point cloud with 2D radiographic images, the PCdare software is run in evaluation mode after the 2D radiographic and 3D PC marker positions and line markings have been saved (subsection 2.1). In evaluation mode, the saved files are reloaded. Then, the biplanar radiographs are positioned in the 3D coordinate system of the EOS system by using the information from the DICOM tags to transform the images from pixel space to a millimeter-based coordinate system. The 2D marker selections from both radiographs are projected orthogonally into 3D (Figure 2A, green), and the 3D position of each marker is calculated by the intersection of the corresponding 3D projections. The ISL in 3D is calculated by the intersection of the projections of the 2D line markings from both radiographs. Then, the 3D surface point cloud, the corresponding 3D PC marker positions, and the 3D line markings of the SPL are loaded in the coordinate system of the 3D scanner. All 3D PC marker positions are then registered with the 3D radiographic marker positions using the estimateGeometricTransform3D MATLAB function. This function uses a least-squares algorithm with singular value decomposition (SVD) to determine a rotation matrix and a translation vector (32). The resulting rigid geometric transformation is then used to transform the point cloud, PC marker positions, and SPL into the EOS coordinate system (Figure 2B). Finally, the transformed coordinates of the point cloud, the 3D marker positions, the SPL, and the 3D ISL are saved in another file.

### 2.3 Method of correlating between SPL and ISL

**Figure 3:**
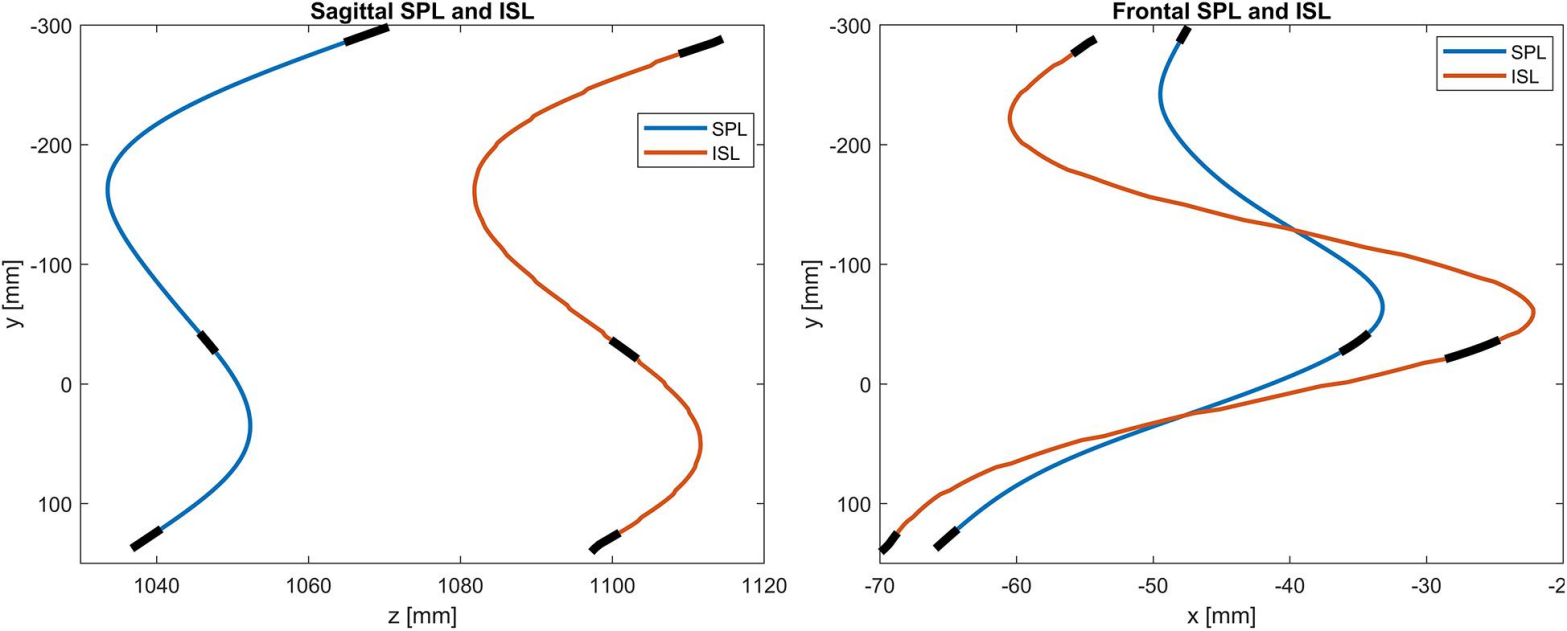
Example of smoothed SPL and ISL for sagittal (left image) and frontal (right image) planes with indication of the positions of C7, T12, and L5 (black). The sagittal plane is spanned by the y and z axes and the frontal plane by the y and x axes of the EOS coordinate system (Figure 2).

The PCdare software can be used in evaluation mode to automatically calculate the correlations between SPL and ISL in the EOS coordinate system. First, the SPL and ISL lines are smoothed with a smoothing spline (MATLAB function: fit from the Curve Fitting Toolbox, Figure 3) and sampled at regular intervals. Then a Procrustes transformation is applied to the ISL (MATLAB function: procrustes). The Procrustes transformation is configured to include only translation and rotation without scaling, which allows the correlation of the shapes to be calculated irrespective of vertical translation and global rotation. The Pearson correlation coefficient (PCC) between SPL and ISL is then calculated separately for the sagittal and frontal planes using the corrcoef MATLAB function to investigate to what degree the shape of the ISL can be estimated from the shape of the SPL using a linear model. The corrcoef MATLAB function calculates the PCC according to Equation 1,

$$\begin{array}{c} PCC(\mathrm{SPL},\mathrm{ISL})=\frac{1}{N-1}\overset{N}{\underset{i=1}{\sum }}\left(\frac{\mathrm{SP}L_{i}-\mu_{\mathrm{SPL}}}{\sigma_{\mathrm{SPL}}}\right)\left(\frac{\mathrm{IS}L_{i}-\mu_{\mathrm{ISL}}}{\sigma_{\mathrm{ISL}}}\right), \end{array}$$ (1)

where N is the number of sampled points, $\mathrm{SP}L_{i}$ is the i-th point of the SPL, $\mu_{\mathrm{SPL}}$ is the mean of all SPL points, $\sigma_{\mathrm{SPL}}$ is the standard deviation of all SPL points; the same definitions apply equivalently to the ISL.

### 2.4 Method of comparing with the current gold standard in clinical practice

**Figure 4:**
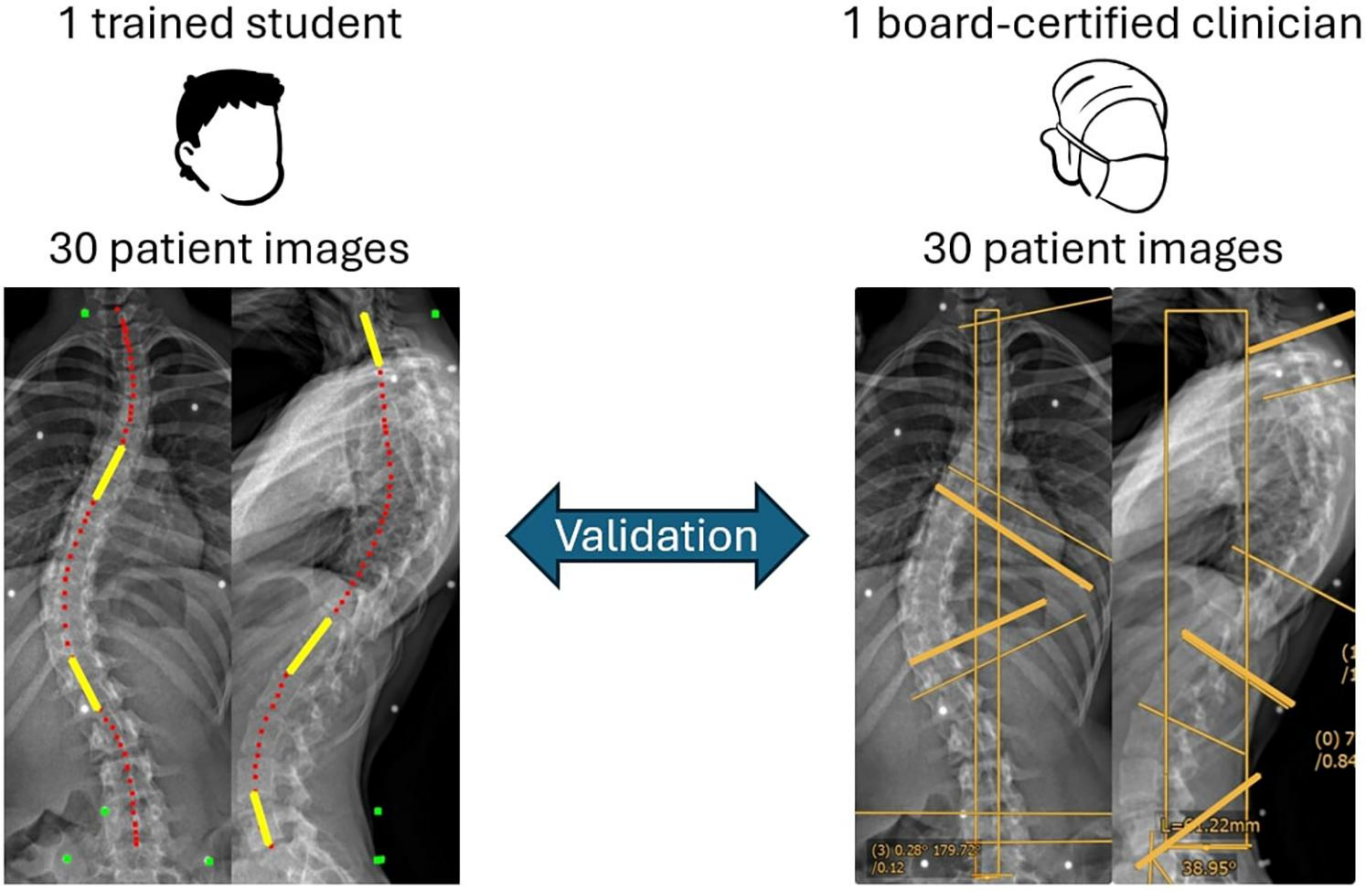
Illustration of validation of cobb angles for frontal, kyphotic, and lordotic curvatures calculated from lines drawn by a HEST student using the PCdare software (left, red) and manually annotated by a board-certified clinician using the current gold standard in clinical practice (PACS, right, yellow). In PCdare the Cobb angles are calculated with local linear regression (left, yellow), in PACS according to the Cobb method.

We used the PCdare software to generate reference lines for the ISL. To validate the reference lines used in research, the Cobb angles calculated from the PCdare software line markings were compared with the gold standard in clinical practice: the Cobb angles annotated in a PACS system. One health science and technology (HEST) student with prior experience using PCdare drew the marker lines for 30 patient images, and one board-certified clinician annotated the same 30 patient images in PACS (MERLIN Diagnostic Workcenter, Version 7.1, Phönix-PACS GmbH, Freiburg im Breisgau, Germany). The patient images were presented chronologically, and the line markings and annotations were made in a single session. The Cobb angles of the frontal, kyphotic, and lordotic curvature were calculated from the smoothed ISL lines with a local linear regression (MATLAB function: fit) at the inflexion points of the curvature (Figure 4, left, yellow) and compared pairwise with the Cobb angles annotated manually by the board-certified clinician (Figure 4, right). The Cobb method was used for frontal (endplates of most tilted vertebrae), kyphotic (T1–T12 endplates), and lordotic (L1–S1 endplates) curvature angles.

## 3 Results

### 3.1 Marker selection and line markings

The evaluation of timestamps of one experienced student revealed an average duration of 2 min and 10 s with a standard deviation (SD) of 27 s to select four markers (C7, L5, PSIS) and draw all line markings (ISL, SPL) from C7 to L5 for a single patient.

### 3.2 3D registration

**Figure 5:**
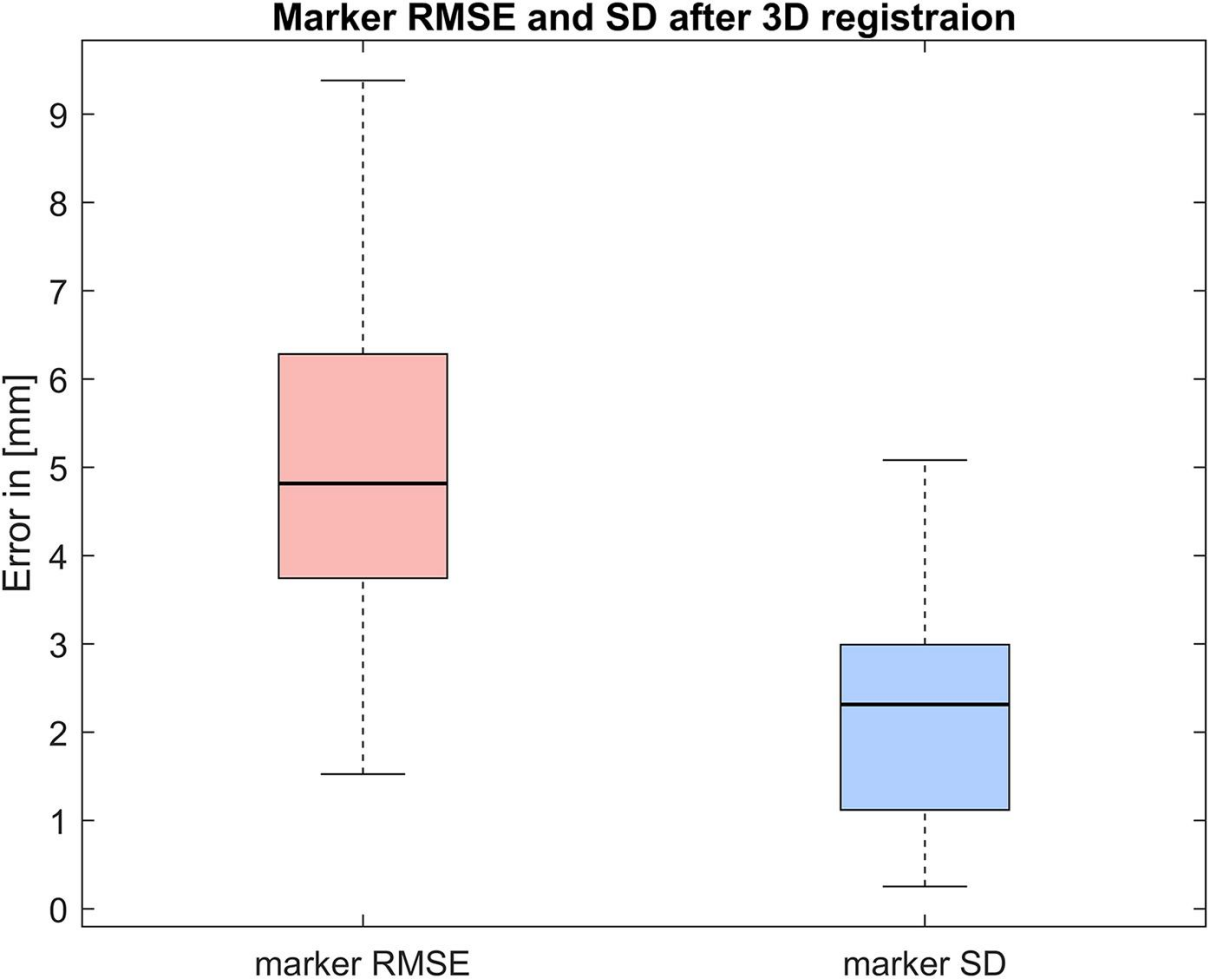
RMSE and SD between back surface scan PC marker positions and 3D radiographic marker positions after 3D registration for 30 patients with four markers each, totaling 120 datapoints.

The discrepancy between the PC marker positions from the back surface scan and the 3D projected marker positions from the radiographic images for 120 datapoints from 30 patients had a median root mean square error (RMSE) of 4.8 mm and median SD of 2.3 mm (Figure 5).

### 3.3 Correlations between SPL and ISL

**Figure 6:**
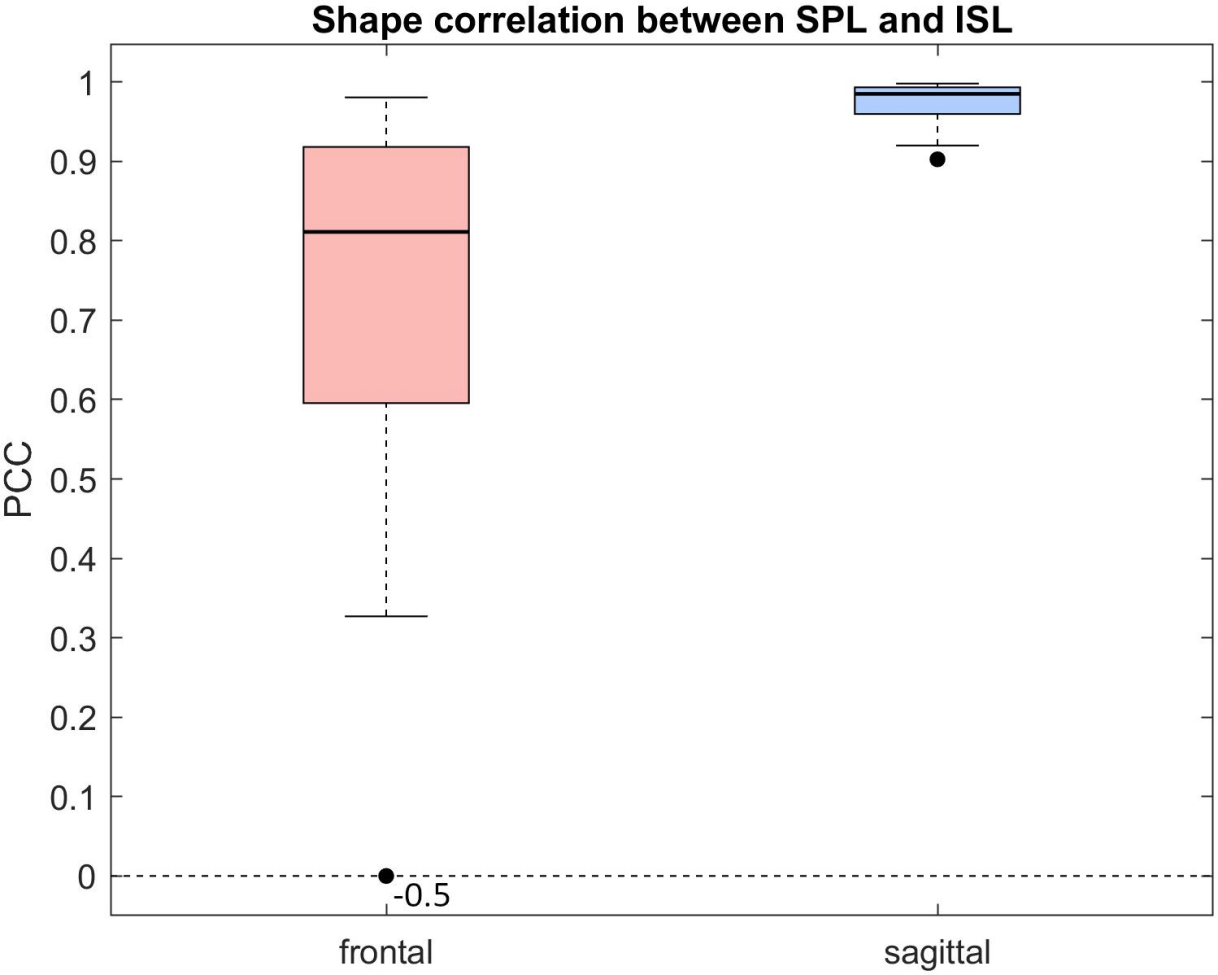
Shape correlation between smoothed SPL and ISL lines for the frontal plane and the sagittal plane after applying a procrustes transformation to data from 30 patients.

The median PCC between SPL and ISL and its interquartile range (IQR) was 0.81 (0.32) for the frontal plane and 0.99 (0.03) for the sagittal plane for 30 patients (Figure 6).

### 3.4 Comparison with the current gold standard in clinical practice

**Figure 7:**
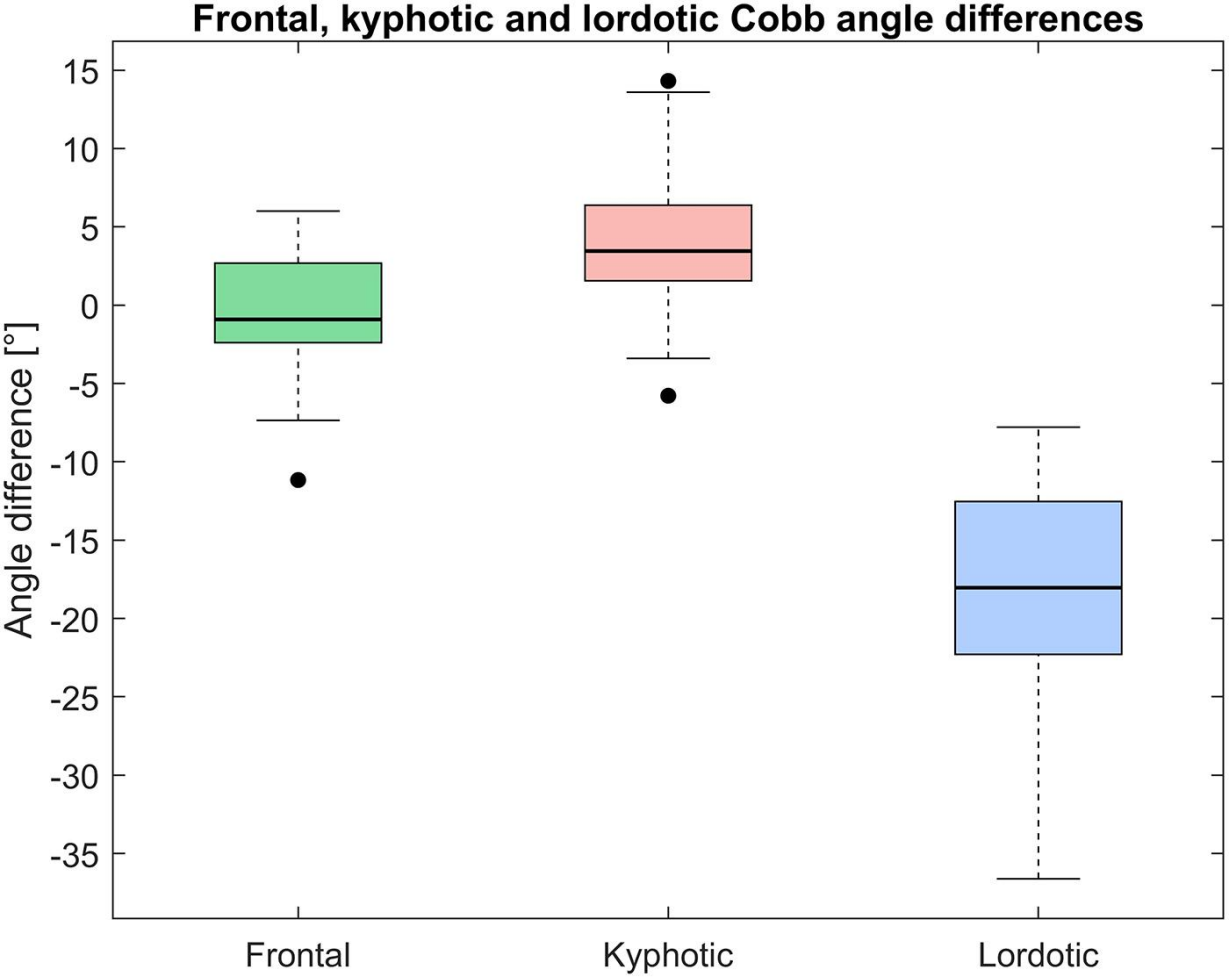
Pairwise difference between cobb angles of frontal, kyphotic, and lordotic curvature calculated from smoothed ISL lines drawn in PCdare and manually annotated in PACS.

Table 1 shows the median Cobb angles and IQR of frontal, kyphotic, and lordotic curvature calculated from the smoothed ISL lines and manually annotated in PACS for 30 patients. The pairwise differences between PCdare and PACS are shown in Table 1 and Figure 7.

**Table 1:** Median cobb angles (IQR) of frontal, kyphotic, and lordotic curvature calculated from the smoothed ISL lines (PCdare) and manually annotated in PACS.

Median Cobb angle (IQR)	Frontal	Kyphotic	Lordotic
PCdare	29° (29°)	41° (20°)	40° (15°)
PACS	29° (25°)	36° (13°)	56° (11°)
Pairwise difference	−0.9° (5.1°)	3.5° (4.8°)	−18° (9.8°)

The correlation coefficients of the Cobb angles of frontal and kyphotic curvature calculated from the smoothed ISL lines with manually annotated Cobb angles were excellent (>0.9), and the correlation of the Cobb angles of the lordotic curvature was strong (>0.7) (Table 2).

**Table 2:** Correlation of cobb angles of frontal, kyphotic, and lordotic curvature calculated from smoothed ISL lines with cobb angles manually annotated in PACS.

Correlation [confidence interval]	Frontal	Kyphotic	Lordotic
PCdare—PACS	0.97 [0.93,0.98]	0.93 [0.84,0.97]	0.73 [0.50,0.87]

## 4 Discussion

The current gold standard in clinical practice evaluates scoliosis from radiographic imaging. This involves radiation exposure for patients and requires time-consuming manual work for clinicians involved in research projects with many patients (33–35). Software for annotating radiographic images (36) can still require several minutes, especially when loading and switching between multiple images and patients. 3D scanning in combination with less frequent radiography can decrease radiation exposure and fully automatic software can reduce evaluation time.

However, 3D scanning is not yet a clinical standard, and as a result, software for processing radiographic images such as PACS, Surgimap, and sterEOS do not allow processing of 3D surface scans. In general, research software to evaluate radiographs together with 3D surface scans is not available. The PCdare software provides semi-automatic registration of 3D surface scans with radiographic images, including evaluation of the relationship between 3D surface and internal modalities for research. PCdare supports any 3D scan in STL or PCD format, and although any resolution can theoretically be used, the authors recommend maintaining high scan accuracy as discussed in (37). Some parts are still manual, but future work includes the automation of the entire pipeline. However, with an evaluation time of about 2 min per patient, the current version already requires less time than PACS, Surgimap, and sterEOS, where in our study evaluation times per patient ranged from 10 min for PACS and Surgimap to at least 30 min for sterEOS. Furthermore, the combined evaluation of radiographs and 3D scans is already fully automatic. Currently, the greatest limitation is the need for markers for registration. Future work includes the automatic detection of inherent landmarks and the implementation of markerless approaches to registering the two modalities.

One advantage of using markers for registration is the straightforward calculation of a simple error metric. The authors identified only a single publication presenting a similar use case (marker-based motion capture) with an average error between registered markers reported at 4.7 mm (38). While our results closely align with this value, this comparison should be approached with caution, as the use cases are not identical. In our study, much of the RMSE is likely attributable to the patient transitioning between rooms. The study protocol was specifically designed to minimize postural errors, and the resulting error aligns with expectations considering this fact. Future work includes further exploration of postural alignment and sources of error.

Our investigation of the relationship between back shape and internal spinal alignment is limited to linear correlation between the spinous process line and the internal spinal alignment. The median correlations between SPL and ISL are strong to excellent, suggesting that linear statistical shape models can estimate ISL from SPL. However, individual estimates can deviate significantly. The correlation results presented in this work suggest that optical 3D scanning should only be integrated into clinical practice alongside less frequent radiography. Moreover, optical approaches have limitations that need to be considered, particularly regarding body weight, where obese subjects are often excluded from scoliosis analysis using optical techniques. In our study, patients mostly had a normal BMI. Correlations across different BMI categories should be examined using a larger and more diverse dataset. It is also important to note that this method cannot fully replace radiography. Its use is limited to specific conditions, and radiographs remain essential for diagnosis and critical decision-making. However, optical approaches remain promising with future implementations including additional information from e.g., asymmetry maps (11, 39–41), from which the asymmetry of the entire back shape can be compared with the corresponding frontal Cobb angle. Future work includes direct evaluations of nonlinear asymmetry approaches and comparison with not only the frontal Cobb angle but also the internal spinal alignment. Additionally, investigating Spearman correlations may reveal whether nonlinear models provide more accurate estimates.

The comparison of PCdare with manual annotation in PACS, showed that the calculation of frontal Cobb angles from lines drawn by an experienced student produced valid results. Furthermore, another study showed that PCdare is fast to use, requires no special training, and that clinicians and students can draw ISL lines very reliably (29). The difference between frontal Cobb angles calculated with PCdare and manual annotation in PACS is within the range of 1.0°–7.2° reported in the literature (36, 42–49). The difference between kyphotic Cobb angles was higher than the difference between frontal Cobb angles, but still within the range reported in the literature. The spine is less clearly visible on lateral radiographs from EOS low dose imaging, especially in the thoracic region (Figure 1), which could explain the higher error values. The median difference and IQR between the lordotic Cobb angles were not within the range reported in the literature. The Cobb angle of the lordotic curvature was calculated in PCdare using a smoothed ISL ending at the inferior endplate of L5. In PACS, the superior endplate of S1 was used. This could explain the systematic error and should be investigated further.

The generation of reference lines for the ISL demonstrate the potential of the PCdare software and possible applications in research. Ongoing work in our lab includes the use of these reference lines for the validation of ISL which are estimated solely from the optical 3D back surface scan. An application example, not presented in this paper but already implemented in PCdare, is the evaluation of the postural alignment between 3D back surface scanning and radiographic imaging from the SPL. Other applications to validate the quality of the study data and protocol include the comparison of other internal structures with the back surface, such as the palpation of spinous processes and positioning of markers. In this study, marker placement served only for registration purposes and was not used to identify anatomical landmarks. Ongoing work in our lab includes the evaluation of three studies, including an inter- and intrarater reliability (IIR) study in which multiple clinicians and students rate the same images multiple times using the PCdare software.

In summary, the PCdare research software results in considerable time savings over manual annotation, especially when evaluating large quantities of patient data. It is publicly available, allows evaluation of the relationship between 3D surface and internal modalities, and has potential for wider application in research. Further automation efforts will improve reliability and increase time savings. With the future addition of markerless and fully automated registration approaches, the PCdare software may be applied wherever registration is required between 3D surface scans and 2D radiographic images.

## 5 Conclusion

We have presented the PCdare research software, which can be used for semi-automatic registration of 3D surface scans with corresponding biplanar radiographs. This allows the relationship between the shape of the back and the internal alignment of the spine to be evaluated in patients with scoliosis. In addition, PCdare can be used to generate reference lines for the internal spinal alignment. Frontal Cobb angles calculated from the ISL with PCdare were comparable to those annotated by a board-certified clinician in PACS, which is the current gold standard in clinical practice.

Further research applications of PCdare include examining the quality of study protocols, such as evaluating postural alignment between 3D back surface scanning and radiographic imaging using the SPL. Future work includes automating the detection of markers and line markings in both the optical 3D scans and the radiographic images. Furthermore, the calculation of Cobb angles of lordotic curvature needs to be improved. The software and code are publicly available (see Data Availability Statement) to facilitate further research into the relationship between the internal alignment of the spine and the external shape of the back with the vision of integrating optical 3D surface scanning into clinical practice for scoliosis applications, aiming to reduce the frequency of radiation exposure.

## Data Availability

The datasets presented in this study can be found in online repositories. The names of the repository/repositories and accession number(s) can be found below: https://github.com/mkaisereth/PCdareSoftware.
